# Practical Notes on Diagnosis and Treatment

**Published:** 1909-08-21

**Authors:** 


### Practical Notes on Diagnosis and Treatment.

### The Operation of Prostatectomy.

If a patient near 70 years has and has had for some time well marked symptoms; if the prostate on rectal examination presents a bi-lobed bulging and is not hard; and if the organ examined bimanually under an anæsthetic is large: then operation is indicated. On the other hand, if the patient is near 60, and has had symptoms only a short time; if the prostate is small both to rectal and bimanual examination: then I am inclined to hold my hand.—*Mr. Cuthbert Wallace.*

### Lichen Plano-Filaris.

This is a very stubborn lesion to treat, but a remarkably good result has been reported when salicylic ointment was applied externally and a mixture containing phosphoric acid and strychnine was given by the mouth. In certain cases of lichen planus, also, benefit follows the administration of phosphoric acid, even when the remedies usually recommended have failed.—*Dr. Agnes F. Savill.*

### Preparation for Curettage.

Curettage should be carried out with the most scrupulous antisepsis. The chief point in an aseptic curettage is the preparation of the vulva and vagina by scrubbing each with sterile wool swabs with ether soap followed by lysol solution. No amount of douching will make the vagina aseptic, for the fluid simply runs in and out without reaching the folds. After the operation no daily douching should be permitted, for this does no good, and may be a potent source of re-infection of the uterus.—*Dr. T. G. Stevens.*

### Calomel in Nephritis.

There is a prevailing impression that mercurial preparations should not be prescribed in cases of kidney disease. It is probable that this fear is overstated. Provided that the patient is closely watched and that every 24 to 48 hours a saline purgative is administered, calomel is often effectively prescribed for the œdema of chronic parenchymatous nephritis.

### Infantile Diarrhœa.

Apart from the acute summer diarrhœa of infants many infants and children suffer in the warmer months of the year from a simple though troublesome form of looseness of the bowels. After a preliminary dose of castor oil it is sufficient to order a diet of boiled milk and thin arrowroot, while for medicine nothing is better than 1 to 3 grains of zinc oxide with a few minims of paregoric given in some syrup and mucilage.

### Painless Treatment of Boils and Carbuncles.

Apply a pledget of cotton soaked in carbolic glycerine, and cover with gutta percha tissue and a bandage. As soon as pus shows gently turn back the epithelium and re-apply the carbolic glycerine. Upon the appearance of a slight cavity introduce some of the glycerine by means of a syringe and again cover as above described. In two or three days the slough separates, and after one final poultice of glycerine the cavity speedily closes, with the minimum of scar, under any simple dressing.—*Dr. A. Ogier Ward.*

